# Consolidating Emergency Department-specific Data to Enable Linkage with Large Administrative Datasets

**DOI:** 10.5811/westjem.2020.8.48305

**Published:** 2020-10-27

**Authors:** Krislyn M. Boggs, Maranatha M. Teferi, Janice A. Espinola, Ashley F. Sullivan, Kohei Hasegawa, Kori S. Zachrison, Margaret E. Samuels-Kalow, Carlos A. Camargo

**Affiliations:** Massachusetts General Hospital, Department of Emergency Medicine, Boston, Massachusetts

## Abstract

**Introduction:**

The American Hospital Association (AHA) has hospital-level data, while the Centers for Medicare & Medicaid Services (CMS) has patient-level data. Merging these with other distinct databases would permit analyses of hospital-based specialties, units, or departments, and patient outcomes. One distinct database is the National Emergency Department Inventory (NEDI), which contains information about all EDs in the United States. However, a challenge with merging these databases is that NEDI lists all US EDs individually, while the AHA and CMS group some EDs by hospital network. Consolidating data for this merge may be preferential to excluding grouped EDs. Our objectives were to consolidate ED data to enable linkage with administrative datasets and to determine the effect of excluding grouped EDs on ED-level summary results.

**Methods:**

Using the 2014 NEDI-USA database, we surveyed all New England EDs. We individually matched NEDI EDs with corresponding EDs in the AHA and CMS. A “group match” was assigned when more than one NEDI ED was matched to a single AHA or CMS facility identification number. Within each group, we consolidated individual ED data to create a single observation based on sums or weighted averages of responses as appropriate.

**Results:**

Of the 195 EDs in New England, 169 (87%) completed the NEDI survey. Among these, 130 (77%) EDs were individually listed in AHA and CMS, while 39 were part of groups consisting of 2–3 EDs but represented by one facility ID. Compared to the individually listed EDs, the 39 EDs included in a “group match” had a larger number of annual visits and beds, were more likely to be freestanding, and were less likely to be rural (all P<0.05). Two grouped EDs were excluded because the listed ED did not respond to the NEDI survey; the remaining 37 EDs were consolidated into 19 observations. Thus, the consolidated dataset contained 149 observations representing 171 EDs; this consolidated dataset yielded summary results that were similar to those of the 169 responding EDs.

**Conclusion:**

Excluding grouped EDs would have resulted in a non-representative dataset. The original vs consolidated NEDI datasets yielded similar results and enabled linkage with large administrative datasets. This approach presents a novel opportunity to use characteristics of hospital-based specialties, units, and departments in studies of patient-level outcomes, to advance health services research.

## INTRODUCTION

The American Hospital Association (AHA) and the Centers for Medicare & Medicaid Services (CMS) each provide important data for health services researchers. Specifically, the AHA Annual Survey database contains hospital-level data, including the number of beds.^1^ CMS maintains several “Hospital Compare” datasets with hospital metrics, in addition to a claims-level dataset that includes information about patient visits.^2^ Merging these data with other datasets would permit novel analyses of the relationship between individual hospital-based specialties, units, and departments, and patient outcomes.

That said, it is not clear whether this type of merge is possible. Focusing on emergency departments (ED), a potential dataset for merging with AHA and CMS is the National Emergency Department Inventory (NEDI).^3^ NEDI collects information about basic ED characteristics, including total and child visit volumes. NEDI lists all EDs in the United States individually. By contrast, AHA and CMS list some EDs individually but group others by hospital network, and they exclude some EDs completely (eg, all autonomous freestanding EDs [FSED]).^4^ As more EDs become part of larger hospital networks, the more likely they are to become grouped in AHA or CMS over time. Because of potential differences between grouped and ungrouped EDs, consolidating data for this merge may be preferential to simply excluding grouped EDs. Our two objectives were to consolidate department-specific (ED) data to enable linkage with AHA and CMS datasets and to determine the effect of excluding grouped EDs on ED-level summary results.

## METHODS

Using the 2014 NEDI-New England database, we identified all 195 New England EDs open that year. We sent a three-page survey to all EDs to obtain more facility data, including information about basic characteristics (eg, visit volumes) and staffing (Supplemental Material). We mailed a hardcopy of the survey up to three times and then contacted non-responding EDs by phone to administer the survey by interview. The number of ED beds, annual number of ED visits, and 24/7 consultant availability were obtained through this survey. FSED status,^4^,^5^ rural location, and academic status were obtained from publicly-available sources, as part of ongoing NEDI-USA database maintenance.^3^ The Partners Human Research Committee classified this project as exempt.

To link NEDI-New England with other datasets, we individually matched NEDI EDs with corresponding EDs in the 2014 AHA and CMS Provider of Services files. We determined that an ED was listed in both datasets if the names and addresses matched exactly. In instances where either differed, we confirmed the match by investigating the ED’s website or calling the ED about the discrepancy. Furthermore, CMS lists all facilities that have ever had an identification number in their annual Provider of Services dataset. This CMS dataset includes EDs that are closed, provider numbers that are no longer active, and facilities without EDs.^2^ This has led to instances where multiple facilities with similar names are listed under a single address. Thus, to only view EDs with active CMS ID numbers in 2014, we filtered by provider category subtypes of “Short Term”, “Children’s” or “Critical Access Hospitals,” and “Active.”

When an ED was not individually listed in AHA or CMS but was affiliated with another listed ED, we considered this a “group match.” We confirmed these affiliations by reviewing a hospital/ED’s website. In instances when an FSED was part of a group match, we used NEDI to further confirm that it was grouped with the appropriate listed parent hospital. Thus, each group included one listed ED and at least one unlisted ED.

Within each group, we consolidated individual ED data to create a single observation, based on calculated totals (eg, number of ED beds) or visit volume-weighted averages of binary responses (eg, rural location). We converted categorical variables into separate binary variables to apply the same visit-volume weighting (Supplemental Material). If the listed ED in a group responded to the NEDI survey, we included that group’s data in the consolidated dataset. We then created two versions of the consolidated dataset: one where final, weighted values were rounded to the nearest integer, and a second where values were unrounded. We used chi square, Fisher’s exact, and Wilcoxon rank-sum tests, as appropriate, to compare NEDI variables in the ungrouped vs grouped EDs and the consistency of results from the original vs consolidated NEDI datasets.

## RESULTS

Of all 195 New England EDs, 169 (87%) completed the NEDI survey. Among these, there were 130 (77%) EDs individually listed in both AHA and CMS. The remaining 39 EDs were part of 21 groups consisting of 2–3 EDs but represented by one facility ID number. There were no instances where a NEDI ED was part of a group in AHA but ungrouped in CMS. Comparing NEDI-New England responses between 130 ungrouped EDs and the 39 grouped EDs, the grouped EDs had a larger number of annual visits and beds. They also were more likely to be FSEDs, more likely to have access to pediatricians, and less likely to be rural (all P <0.05, Table 1). The ungrouped and grouped EDs did not differ by academic status, nor by their access to ED consultants other than pediatricians (eg, psychiatrists, surgeons).

Two grouped EDs were excluded because the listed ED in the group did not respond to the original NEDI survey; the remaining EDs were consolidated into 19 observations. Specifically, these 19 observations represented 41 total EDs: 19 EDs that were listed in AHA and CMS and completed the NEDI survey; 18 EDs grouped with an AHA- and CMS-listed ED that completed the NEDI survey; and four EDs that did not complete the NEDI survey but that were grouped with an AHA- and CMS-listed ED that did.

The consolidated dataset contained 149 observations representing 171 EDs. Both the rounded and unrounded consolidated datasets yielded aggregated results that were similar to those of the 169 responding EDs (Table 2). For example, 12% of 169 EDs were in rural areas vs 14% of 149 observations in both the rounded and unrounded consolidated datasets. Likewise, 7% of 169 EDs were academic vs 6–7% of observations in the rounded and unrounded consolidated datasets. Finally, the median annual total visit volumes of responding EDs and observations were also similar (30,000 versus 32,398 visits, respectively).

## DISCUSSION

Using EDs as an example, our study shows that it is possible to consolidate individual hospital-based data to enable linkage with large administrative datasets, and that this method preserves the integrity of the original dataset better than the alternative method of excluding grouped EDs. Excluding all grouped EDs would result in the omission of 23% of collected data (39/169 EDs). Our consolidation methods, however, preserved most of the data from these EDs, with only 1% of collected data omitted (2/169 EDs). We found that the consolidated and original datasets yielded similar results, but excluding all grouped EDs would have resulted in a biased dataset. For example, compared to ungrouped EDs, the grouped EDs had more visits and beds, and were less likely to be rural. Since the rounded and unrounded values in the consolidated dataset yielded similar aggregated results, we propose using the rounded consolidated dataset going forward, which better reflects the variable type of the original, granular dataset. These methods may also be applicable to the linkage of datasets of other individual, hospital-based specialties, units, and departments within administrative datasets.

While prior research and methods favor the use of publicly-available AHA or CMS datasets,^6^–^12^ our results demonstrate that the exclusion of EDs in those datasets may lead to information bias. Most clearly, none of the FSEDs included in NEDI are individually listed in AHA or CMS. While FSEDs make up only 4% of all responding New England EDs open in 2014, the number of FSEDs has increased sharply since then, both in New England and even more so on a national level.^13^ For example, as of 2017 FSEDs made up 12% of all US EDs, 4 and as of August 2020 there were 684 total FSEDs open in the US (unpublished data). Since all New England FSEDs were part of groups, excluding them completely would have disregarded an increasingly important provider of emergency care.

Furthermore, given that EDs that were part of groups were all also part of hospital networks, we would anticipate that an increase in health networks would result in an increase in EDs requiring grouping in future datasets, especially given that hospital and health system mergers increased in the years leading up to and after 2014, peaking in 2017. 14 This increase conveys that these methods may perhaps be of increasing importance going forward. Further supporting this observation is that the number of facilities listed in AHA have decreased each year since 2008,^15^ whereas the number of individual EDs in NEDI has increased each year since 2001,^16^ suggesting that although EDs continue to open, the increase in number of EDs in health networks leads to a lower number of facilities individually listed in AHA.

## LIMITATIONS

The NEDI-New England survey relies on self-reported results. However, we mitigated this limitation by obtaining facility data from the ED director, who presumably is the most knowledgeable person about the operations of his or her ED. Also, our consolidation methods still required that among groups where not all EDs completed the survey data had to be dropped if the listed ED did not participate. However, this resulted in minimal data loss among responding New England EDs, with only two (1%) having dropped data. Finally, the consolidation of ED-specific data for linkage may introduce bias. However, we believe this bias is limited, given that the data of most EDs are preserved during this process, which improves the overall representativeness of the dataset. Furthermore, the consolidated and granular results are similar.

## CONCLUSION

ED-specific data can be consolidated to enable linkage with large administrative datasets in a way that maintains the integrity of the original data. Excluding all grouped EDs would have resulted in a smaller, non-representative dataset. In contrast, the original vs consolidated NEDI datasets yielded similar results. We propose using the rounded consolidated dataset to better reflect the variable type of the original, granular dataset. This novel approach presents an opportunity to use characteristics of hospital-based specialties, units, or departments in studies of patient-level outcomes, to advance health services research.

## Supplementary Information





## Figures and Tables

**Table 1:** Characteristics of emergency departments that were not part of groups and parts of groups among National Emergency Department Inventory–New England survey responders, n = 169.

	EDs not in groups n=130	EDs part of groups n=39	

Basic ED characteristics	n (%)	n (%)	P-value
Freestanding ED	0 (0)	6 (15)	<0.001
Rural location	21 (16)	0 (0)	0.004
Academic	6 (5)	5 (13)	0.13
Number of ED beds, median (IQR)	20 (10–30)	29 (15–42)	0.01
Annual # of vistis, median	27,900 (14,000–50,000)	41,019 (20,310–57,000)	0.03

ED staffing, timing of consultants	n (%)	n (%)	P-value

Consutant availability 24/7			
Anesthesiologist	112 (86)	29 (74)	0.08
Cardiologist	78 (60)	29 (74)	0.10
General surgeon	111 (85)	30 (77)	0.21
Neurologist	58 (45)	22 (56)	0.20
Obstetrician-gynecologist	106 (82)	26 (67)	0.08
Pediatrician	81 (62)	17 (44)	0.04
Plastic surgeon	23 (18)	10 (26)	0.27
Psychiatry	45 (35)	11 (28)	0.46

*ED*, emergency department, *IQR*, interquartile range; *24/7*, 24 hours per day and 7 days per week.

**Table 2:** Emergency department characteristics based on all responses and based on consolidated datatset, among National Emergency Department Inventory–New England survey responders.

	All NEDI respondents n=169 EDs	Rounded responses based on consolidated dataset n=149 observations*	Difference in % or medians	Unrounded responses based on consolidated dataset n=149 observations*	Difference in % or medians

Basic ED characteristics	n (%)	n (%)		n (%)	
Freestanding ED	6 (4)	1 (1)	3	1 (1)	3
Rural location	21 (12)	21 (14)	−2	21 (14)	−2
Academic	11 (7)	10 (7)	0	9 (6)	1
Number of ED beds, median (IQR)	22 (11–33)	23 (11–37)	−1	23 (11–37)	−1
Annual # of vistis, median	30,000 (16,000–51,000)	32,398 (15,650–57,000)	−2,398	32,398 (15,650–57,000)	−2,398

ED staffing, timing of consultants	n (%)	n (%)		n (%)	

Consutant availability 24/7					
Anesthesiologist	141 (83)	129 (87)	−4	128 (86)	−3
Cardiologist	107 (63)	97 (65)	−2	95 (64)	−1
General surgeon	141 (83)	130 (87)	−4	128 (86)	−3
Neurologist	80 (47)	73 (49)	−2	71 (48)	−1
Obstetrician-gynecologist	132 (78)	123 (83)	−5	121 (81)	−3
Pediatrician	98 (58)	93 (62)	−4	92 (62)	−4
Plastic surgeon	33 (20)	30 (20)	0	29 (19)	1
Psychiatry	56 (33)	53 (36)	−3	52 (35)	−2

* 149 observations represent 171 individual EDs: 19 EDs that completed the NEDI survey and that were listed in AHA and CMS, 18 EDs that completed NEDI survey and grouped with an ED individually listed in AHA and CMS, and 4 EDs that did not complete NEDI survey but that were grouped with an ED listed in AHA and CMS that did.

*NEDI*, National Emergency Department Inventory; *ED*, emergency department; *IQR*, interquartile range; *24/7*, 24 hours per day and 7 days per week; *AHA*, American Hospital Association; *CMS*, Centers for Medicare and Medicaid Services.
