# Supplementary material for: Consolidating Emergency Department-specific Data to Enable Linkage with Large Administrative Datasets
Source: West J Emerg Med. 2020 Oct 27;21(6):141–5. doi: 10.5811/westjem.2020.8.48305 (PMC7673880; doi:10.5811/westjem.2020.8.48305)
Supplement: Supplementary file 1 [file wjem-21-141-s001.docx]

**SUPPLEMENTAL MATERIAL**

**Creation of Consolidated 2014 NEDI-New England Dataset**

This document describes consolidation of individual variables, by variable type. The examples below assume that “ED A” and “ED B” are individual EDs that must be consolidated into a single observation.

1. **When the variable is a total number, all individual ED numbers are summed for a consolidated total.**

For example, if ED A’s total visit volume is 10,000, and ED B’s total visit volume is 950, the consolidated total visit volume would be 10,950.

Survey questions consolidated under this rule: A4(a,b), A5, A6a, A10, B1(a-d)

1. **When the variable is a percent of a total, we calculated the consolidated percent based on a visit volume-weighted average.**

For example, if ED A reports that 10% of patients arrive to the ED by ambulance and ED B reports that 50% of patients arrive to the ED by ambulance, we calculate that 1,000 patients (10,000 * 10%) arrive by ambulance to ED A, and 475 arrive by ambulance to ED B. In total, 1,475, or 13.5% of patients (1,475 arrivals by ambulance / 10,950 total ED visits), arrive by ambulance.

Survey questions consolidated under this rule: A7-A9, B3, E3

1. **When the variable is binary, a visit volume-weighted average of responses is calculated, and then the final value is rounded to again yield a binary variable.**

For example, if ED A reports an attending on duty in the ED 24/7, and ED B reports that an attending is NOT on duty 24/7, we weight these responses as follows: (1 * 10,000 visits) + (0 * 950 visits) = 10,000. Then, we calculate 10,000/10,950 = .913, which rounds to 1 (yes).

Survey questions consolidated under this rule: A2, A6b, B2, B2a(i,ii), B4, C1, C4(a-g), C5, C6, D1(a-k, 1^st^ and 3^rd^ subquestions only), E1(a,b), E4, E4a, F1(a-e, both subquestions), F2, G1(a-e), rural status, academic ED status, FSED status

1. **When the variable is categorical and non-ordinal, we created a separate, binary variable for each response option. To consolidate each of these binary variables, we followed the rules outlined in rule #3 above.**

For example, question E5 asks the respondent to describe their hospital’s ED as “Under capacity”, “Good balance”, “At capacity”, or “Over capacity”. If ED A chooses “At capacity” and ED B chooses “Over capacity”, we weight these responses as follows:

Under capacity = 0, no (neither ED selected this option)

Good balance = 0, no (neither ED selected this option)

At capacity = 1, yes ([1 * 10,000] + 0 [950] = 10,000. Then, we calculate 10,000/10,950 = .913, which rounds to 1)

Over capacity = 0, no ([0 * 10,000] + [1 * 950] = 950. Then, we calculate 950/10,950 = .087, which rounds to 0)

Survey questions consolidated under this rule: C3(a-h), E2, E5

1. **When the variable is ordinal, we first re-assign each ranged response option as the midpoint of the range. The two exceptions are:**

- **The response option “>60 minutes”, which we reassign as “75 minutes”**
- **The response option “>6 hours”, which we reassign as “7.5 hours”**

**We then take the visit volume-weighted averages of these assigned options, and re-categorize them based on the original, ranged response options.**

For example, if ED A reports that, on average, it takes 0-29 minutes for an anesthesiologist to arrive in-person to the ED, and ED B reports that, on average, it takes 30-59 minutes, we consolidate these responses as follows: First, we re-code ED A’s response to 14.5 minutes and ED B’s to 44.5. We then take the weighted average of these responses: (14.5 * 10,000) + (44.5 * 950) = 182,275. Then, we calculate 182,275/10,950 = 16.6. Finally, we re-categorize this weighted average back into the appropriate original, ranged response option: 0-29 minutes.

Survey questions consolidated under this rule: C7, D1 (2^nd^ subquestion only), D2 (a-j)

For all above variables, “not applicable” responses are not included when calculating weighted averages. If the listed hospital in a group does not respond to the question, we drop data for the entire group for that specific variable. If an unlisted hospital in a group does not respond, we still include data for the group for that specific variable.
