# Supplementary material for: Consolidating Emergency Department-specific Data to Enable Linkage with Large Administrative Datasets
Source: West J Emerg Med. 2020 Oct 27;21(6):141–5. doi: 10.5811/westjem.2020.8.48305 (PMC7673880; doi:10.5811/westjem.2020.8.48305)
Supplement: Supplementary file 2 [file wjem-21-141-s002.pdf]

Please answer questions with estimates for the year **2014** (unless otherwise indicated)

A. BASIC CHARACTERISTICS

A1. In 2014, was your ED open:

a. 24 hours/day, 7 days/week? ☐1 Yes ☐0 No

b. 365 days per year? ☐1 Yes ☐0 No

If NO to either question**,** or if there was a change in 2014, please explain: ______________________________

A2. Although every ED would evaluate an emergent patient of any age, some EDs predominantly see older patients (e.g., age 12+ years). Other EDs, often in children's hospitals, may predominantly see younger patients (e.g., age <18 years). Does your ED have an age-related policy?

☐1 Yes (specify age range): ____________ ☐0 No, all ages seen

A3. For EDs that regularly treat adults: **Does your ED have a separate "pediatric ED" (i.e., a dedicated ED area for children only)?**

☐1 Yes ☐0 No ☐8 Not applicable (e.g., children's hospital)

A4. a. Number of ED Beds (exclude hallway and ED-based OBS unit, if applicable) # ED BEDS [ ][ ][ ]

b. Number of ED-based OBS unit beds # OBS BEDS [ ][ ][ ]

A5. Please indicate the total number of patient visits at your ED and the 12 month reporting period to which they apply.

ED VISITS [ ][ ][ ][ ][ ][ ] **From** ____/____ (MM YY) **to** ____/____ (MM YY)

A6. Approximate number of visits by children per year:

a. Children (e.g., age <18) [ ][ ][ ][ ][ ][ ]

b. If your ED uses an older age to distinguish between children and adults (e.g., age 21), and it's difficult to obtain data for age <18, please respond above according to your ED cut-off (specify cut-off: **age <** _____ **years**)

A7. Approximate percent of patients that arrived to the ED by ambulance % BY AMBULANCE [ ][ ]

A8. Approximate percent of ED patients that were uninsured or 'self-pay' % UNINSURED [ ][ ]

A9. Approximate percent of all ED visits that led to admission, including ICU and OBS admissions (ED-based OBS unit + OBS status) % ADMITTED [ ][ ]

A10. Approximate number of critical care transfers to other facilities (i.e., transfers requiring a level of care beyond ALS – Advanced Life Support) # PATIENTS/YR [ ][ ][ ][ ]

B. STAFFING

B1. For 2014, please indicate the average total number of total staff, after accounting for departures and new hires. Totals are expressed as full-time employees (FTE) – e.g., two half-time = 1 FTE.

a. # FTE ED attending physicians [ ][ ][ ]

b. # FTE ED nurses [ ][ ][ ]

c. # FTE ED physician assistants [ ][ ][ ]

d. # FTE ED nurse practitioners [ ][ ][ ]

B2. Is at least one attending physician (not resident) on duty in the ED 24 hours/day, 7 days/week? (exclude on-call physicians)

☐1 Yes (Go to B3) ☐0 No (Go to B2a)

B2a. When a physician is unavailable, is any physician available to the ED by two-way voice communication 24 hours/day, 7 days/week:

i) From within your hospital? ☐1 Yes ☐0 No

ii) From outside of your hospital? ☐1 Yes ☐0 No

B3. In 2014, percent of attending emergency physicians board-certified or board-eligible (BC/BE) by ABEM, AOBEM or ABP/Peds EM % BC/BE [ ][ ][ ]

B4. Is at least one Certified Emergency Nurse (CEN) on duty in the ED 24 hours/day, 7 days/week?

☐1 Yes ☐0 No ☐2 Don't know

B5. Do you have identified coordinators for pediatric emergency medicine in your ED? [check all that apply]

☐1 Yes, physician coordinator(s)
☐2 Yes, nurse coordinator(s)
☐0 No

C. ELECTRONIC RESOURCES IN THE ED

C1. Does your ED obtain consultation via video conferencing equipment? (e.g., video transmission to outside experts for evaluation of an acute stroke patient in your ED) ☐1 Yes (specify): ______________________________ ☐0 No

C2. Is a computer system used to collect real-time clinical data in the ED? ☐1 Yes ☐2 Partial/In Progress ☐0 No

C3. Are the following available electronically in the ED?

**Electronic version of:**	**Yes**	**Partial/ In Progress**	**No**
a. Patient tracking information in ED (e.g., location, admission status)	☐1	☐2	☐0
b. Hospital discharge summaries	☐1	☐2	☐0
c. Current outpatient medications	☐1	☐2	☐0
d. ED visit notes	☐1	☐2	☐0
e. Radiographic images from a prior visit	☐1	☐2	☐0
f. Old electrocardiograms	☐1	☐2	☐0
g. Laboratory results	☐1	☐2	☐0
**If YES:** Automatic notification of critical values	☐1	☐2	☐0
h. Computerized medication ordering (CPOE)	☐1	☐2	☐0
**If YES:** Computerized error checking (e.g., warnings about medication cross-reaction, allergies, dosage)	☐1	☐2	☐0

C4. Please respond "yes" or "no" to the following questions.

	Yes	No
a. Is there a clinical laboratory available for your ED with the capacity to perform a potassium blood test 24/7?	☐1	☐0
b. Is there a cardiac monitor available immediately in the ED?	☐1	☐0
c. Is there a mechanical ventilator available immediately in the ED?	☐1	☐0
d. Is there a respiratory isolation (negative pressure) room available in the ED?	☐1	☐0
e. Is a CT scanner immediately available to the ED?	☐1	☐0
f. Is a MRI scanner immediately available to the ED?	☐1	☐0
g. Is there a radiologist (including tele-radiologist) available 24 hours/day, 7 days/week to read radiographs?	☐1	☐0

C5. Is point-of-care (PoC) ultrasound available immediately in the ED? ☐1 Yes ☐0 No
(PoC ultrasound is also known as "bedside" or "emergency" ultrasound.)

C6. In your ED, do the emergency physicians (not radiologists, cardiologists, etc.) use PoC ultrasound for clinical care? ☐1 Yes ☐0 No

If **NO** to C5 or C6, **SKIP TO D1**
If **YES**, continue

C7. Approximately what percentage of your emergency physicians use PoC ultrasound as part of their clinical practice?
(For example, 40% would mean that 4 out of 10 emergency physicians use PoC ultrasound as part of their clinical practice.)
☐1 1-20% ☐2 21-40% ☐3 41-60% ☐4 61-80% ☐5 81-100%

D. TIMING OF CONSULTATIONS, TESTS AND TRANSFERS

We understand that EDs do not always operate under ideal circumstances. In this survey, we encourage you to be candid about your hospital's ED with the assurance that your responses will be kept completely confidential and reported as aggregate data only.

D1. Are the following consultants available in-person to the ED?

		Yes	No		On average, how long does the consultant take to arrive? 0-29 min	30-59 min	≥ 60 min	Is the consultant available 24 hours/day, 7 days/week? Yes	No
a.	Anesthesiologist	☐1	☐0	If YES →	☐0	☐1	☐2	☐1	☐0
b.	Cardiologist	☐1	☐0	If YES →	☐0	☐1	☐2	☐1	☐0
c.	General Surgeon	☐1	☐0	If YES →	☐0	☐1	☐2	☐1	☐0
d.	Neurologist	☐1	☐0	If YES →	☐0	☐1	☐2	☐1	☐0
e.	Neurosurgeon	☐1	☐0	If YES →	☐0	☐1	☐2	☐1	☐0
f.	Obstetrician-Gynecologist	☐1	☐0	If YES →	☐0	☐1	☐2	☐1	☐0
g.	Orthopedic Surgeon	☐1	☐0	If YES →	☐0	☐1	☐2	☐1	☐0
h.	Pediatrician	☐1	☐0	If YES →	☐0	☐1	☐2	☐1	☐0
i.	Plastic Surgeon	☐1	☐0	If YES →	☐0	☐1	☐2	☐1	☐0
j.	Psychiatrist	☐1	☐0	If YES →	☐0	☐1	☐2	☐1	☐0
k.	Hand Surgeon	☐1	☐0	If YES →	☐0	☐1	☐2	☐1	☐0

D2. What is the average time it takes to obtain the following in the ED? For smaller EDs, some of the tests or equipment listed below may not be available, and some of the other questions may not be applicable. If this is the case, please check the "NA" box.

	< 15 min	15-29 min	30-59 min	≥ 60 min	NA
a. Basic laboratory test results (e.g. CBC, basic chemistry panel)	☐1	☐2	☐3	☐4	☐8
b. Arterial blood gas results	☐1	☐2	☐3	☐4	☐8
c. Electrocardiogram in adult with chest pain	☐1	☐2	☐3	☐4	☐8
d. Chest x-ray for patient with respiratory distress	☐1	☐2	☐3	☐4	☐8
e. Non-contrast head CT scan to rule-out hemorrhagic stroke	☐1	☐2	☐3	☐4	☐8
f. Type O negative blood for emergent transfusion	☐1	☐2	☐3	☐4	☐8
g. Type specific blood for emergent transfusion	☐1	☐2	☐3	☐4	☐8
		< 30 min	30-59 min	≥ 60 min	
h. Time lapsed between request for patient transfer and departure from ED to an intensive care unit (e.g. on a mechanical ventilator)		☐0	☐1	☐2	☐8
i. Time lapsed between request for patient transfer and departure from ED to the Operating Room (e.g. for emergent condition)		☐0	☐1	☐2	☐8
		< 3 hrs	3-6 hrs	> 6 hrs	
j. Time lapsed between request for patient transfer and departure from ED to a psychiatric inpatient bed		☐0	☐1	☐2	☐8

Please answer questions with estimates for the year **2014** (unless otherwise indicated)

E. CROWDING

E1. On a typical day at 6 pm:

a. Are there any ED patients being cared for primarily in the hallway?

☐1 Yes ☐0 No

b. Are there patients that "board" in the ED for >2 hours until an inpatient bed becomes available?

☐1 Yes ☐0 No

E2. While patients are boarding in the ED please indicate the physician of record:

☐1 Inpatient attending
☐2 ED attending
☐3 Other (specify): ______________________

E3. Approximate percent of ED patients that left before being seen (LBBS)

% LBBS [ ][ ]

E4. In 2014, was your hospital ever on ambulance diversion?

☐1 Yes ☐0 No (Go to E5)

If YES,
a. On average, over the entire year, approximately how many hours/month was your hospital on diversion?

DIVERSION HRS/MO [ ][ ][ ]

E5. In 2014, how would you describe your hospital's ED?

☐1 Under capacity ☐2 Good balance
☐3 At capacity ☐4 Over capacity

F. THE CHOOSING WISELY INITIATIVE

F1. Is there a guideline/policy/clinical pathway, or computerized decision support regarding the use of the following in your ED?
Some questions below do not apply to pediatric EDs / children's hospitals, or those without CT/MRI capabilities. If this is the case, please check the "NA" box.

	Guideline/Policy/ Clinical pathway		Computerized decision support		
	**Yes**	**No**	**Yes**	**No**	**NA**
a. Head CT in patients with minor head injury who are at low-risk based on validated decision rules	☐1	☐0	☐1	☐0	☐8
b. Head CT in asymptomatic adult patients with syncope, insignificant trauma, and a normal neuro exam	☐1	☐0	☐1	☐0	☐8
c. CT pulmonary angiography in patients at low-risk of pulmonary embolism and either a negative Pulmonary Embolism Rule-Out Criteria (PERC) score or negative D-dimer	☐1	☐0	☐1	☐0	☐8
d. Lumbar spine MRI in adult patients with non-traumatic back pain, no neurological deficits, and no suspicion of a serious underlying condition (e.g., vertebral infection, cauda equina syndrome, or cancer with bony metastasis)	☐1	☐0	☐1	☐0	☐8
e. Abdominal/pelvic CT in young otherwise healthy patients with a known history of kidney stones who present with symptoms consistent with uncomplicated renal colic	☐1	☐0	☐1	☐0	☐8

F2. Do clinicians receive individual feedback regarding use of advanced imaging (e.g., compared to other clinicians in your ED)? ☐1 Yes ☐0 No

G. OPIOID MANAGEMENT IN ED PATIENTS

G1. Is there a policy to promote the following actions in your ED?

	**Yes**	**No**
a. Use a specific screening tool (1 or more questions) for targeted screening of patients with suspected prescription opioid abuse potential	☐1	☐0
b. Access your state's Prescription Drug Monitoring Program (statewide database of patients' controlled substance prescription histories) before writing a prescription for opioids	☐1	☐0
c. Notify primary opioid prescriber or primary care provider when prescribing opioids for ED patients with chronic pain	☐1	☐0
d. Refer patients with opioid abuse to treatment and recovery resources	☐1	☐0
**If YES:** Is the patient typically referred to any of the following?		
1. Detox or rehabilitation facility	☐1	☐0
2. Social worker / case manager	☐1	☐0
3. Other (specify): ______________________________	☐1	☐0
e. Prescribe or dispense naloxone to patients at risk of opioid overdose after ED discharge	☐1	☐0

Thank you! Please contact [redacted] if you have any questions or comments.
